# Epidemiology and Seasonality of Endemic Human Coronaviruses in South African and Zambian Children: A Case-Control Pneumonia Study

**DOI:** 10.3390/v13081513

**Published:** 2021-07-31

**Authors:** Vicky L. Baillie, David P. Moore, Azwifarwi Mathunjwa, Daniel E. Park, Donald M. Thea, Geoffrey Kwenda, Lawrence Mwananyanda, Shabir A. Madhi

**Affiliations:** 1 Medical Research Council: Vaccines and Infectious Diseases Analytics, University of the Witwatersrand, Johannesburg 2050, South Africa; David.Moore@wits.ac.za (D.P.M.); azwifarwim@nicd.ac.za (A.M.); Shabir.Madhi@wits.ac.za (S.A.M.); 2 Department of Science and Technology/National Research Foundation: Vaccine Preventable Diseases Unit, University of the Witwatersrand, Johannesburg 2050, South Africa; 3 Department of Paediatrics & Child Health, Chris Hani Baragwanath Academic Hospital and University of the Witwatersrand, Johannesburg 1864, South Africa; 4 Department of International Health, International Vaccine Access Center, Johns Hopkins Bloomberg School of Public Health, Baltimore, MD 21205, USA; danpark@email.gwu.edu; 5 Milken Institute School of Public Health, Department of Epidemiology, George Washington University, Washington, DC 20052, USA; 6 Department of Global Health, Boston University School of Public Health, Boston, MA 02118, USA; dthea@bu.edu; 7 Department of Biomedical Sciences, School of Health Sciences, University of Zambia, Lusaka 50110, Zambia; jaffekwenda@gmail.com; 8 Right to Care-Zambia, Department of Global Health, Boston University School of Public Health, Boston, MA 02118, USA; Lawrence.Mwananyanda@righttocare-zambia.org

**Keywords:** coronavirus, epidemiology, childhood, pneumonia

## Abstract

Endemic human coronaviruses (HCoV) are capable of causing a range of diseases from the common cold to pneumonia. We evaluated the epidemiology and seasonality of endemic HCoVs in children hospitalized with clinical pneumonia and among community controls living in countries with a high HIV burden, namely South Africa and Zambia, between August 2011 to October 2013. Nasopharyngeal/oropharyngeal swabs were collected from all cases and controls and tested for endemic HCoV species and 12 other respiratory viruses using a multiplex real-time PCR assay. We found that the likelihood of detecting endemic HCoV species was higher among asymptomatic controls than cases (11% vs. 7.2%; 95% CI: 1.2–2.0). This was however only observed among children > 6 months and was mainly driven by the *Betacoronavirus* endemic species (HCoV-OC43 and –HKU1). Endemic HCoV species were detected through the year; however, in Zambia, the endemic *Betacoronavirus* species tended to peak during the winter months (May–August). There was no association between HIV status and endemic HCoV detection.

## 1. Introduction

Coronaviruses (CoVs) from the family *Coronaviridae* (Order *Nidovirales*) are enveloped, single-stranded, zoonotic RNA viruses [1]. There are four genera of CoVs, two of which—*Alphacoronaviruses* and *Betacoronaviruses*—are capable of infecting humans causing a variety of symptoms, ranging from gastrointestinal to cardiac and respiratory disease. Further, the respiratory disease can range from a common cold to more severe infections, including bronchitis and pneumonia [2,3]. Four endemic human coronaviruses (HCoVs) are commonly detected in humans, namely HCoV-229E, -HKU1, -NL63 and -OC43 [3]. More recently, novel severe acute respiratory syndrome CoV (SARS-CoV) emerged in 2002, Middle East respiratory syndrome CoV (MERS-CoV) in 2012 and the 2019 novel CoV (SARS-CoV-2) is currently causing a global pandemic with symptoms ranging from mild respiratory and gastrointestinal disease to severe pneumonia and death. HCoV-OC43, -HKU1, SARS-CoV, SARS-CoV-2 and MERS-CoV are all part of the *Betacoronavirus* genera and HCoV-229E and NL63 are part of the *Alphacoronavirus* genera [3]. Endemic HCoVs are most commonly detected as co-infections with other respiratory viruses [2,3,4,5] or in immunocompromised children with underlying chronic disease [3,4]. However, limited information is available on the interactions between HCoVs and human immunodeficiency virus type-1 (HIV), and severity of disease.

In this study, we analyzed the prevalence of endemic HCoV species among children < 5 years of age hospitalized with pneumonia, together with age-matched community controls, living in two African countries with high HIV prevalence, namely South Africa and Zambia.

## 2. Materials and Methods

### 2.1. Case and Control Definitions

This study was undertaken in two sites which had a high HIV prevalence, namely Soweto, South Africa (920 cases and 964 controls) and Lusaka, Zambia (617 cases and 686 controls), from August 2011 through January 2014. Details on enrolment of cases and controls, sample testing and clinical evaluation in the Pneumonia Etiology Research for Child Health (PERCH) study have been published [6,7]. Briefly, cases were children hospitalized with World Health Organization (WHO) criteria of clinically defined severe or very-severe pneumonia, and controls were children actively recruited from the same community in which cases resided. Controls were age-frequency, HIV-status and seasonally matched to cases. The controls were analytically stratified into those with respiratory tract infection (RTI), including if they had a cough, runny nose, ear discharge, wheezing or difficulty breathing together with either a fever (temperature ≥ 38 °C in the past 48 h) or a sore throat but absence of signs of severe pneumonia, and those without RTI. This was a cross-sectional study, so cases and controls were sampled, and detailed demographic and clinical data obtained at the time of enrolment into the study. This clinical assessment was how RTI (*n* = 165) and asymptomatic controls (*n* = 1485) were identified.

### 2.2. Specimen Collection and Laboratory Testing

Flocked nasopharyngeal (NP) swab (Flexible minitip, Copan^®^) and rayon oropharyngeal (OP) swab specimens were collected from all cases and controls on enrolment into the study. The swabs were placed together in a vial containing 3 mL of Universal Transport Medium (Copan^®^). The NP/OP specimens were maintained at 4–8 °C for a maximum of 24 h and then archived at −70 °C until tested. Total nucleic acids were extracted from the NP/OP swabs using the NucleiSens EasyMag extraction system as per manufactures instructions (BioMerieux, Marcy l’Etoile, France) and were tested by multiplex real-time PCR (FTD Resp 33, Fast-track Diagnostics, Sliema, Malta) [8]. The FTD Resp 33 panels detected for 19 viruses (HRV (A, B and C not differentiated), Influenza virus (A, B and C separately), PIV (1, 2, 3 and 4 separately), Coronavirus (HKU1, OC43, NL63, 229E separately), Bocavirus, Human Metapneumoviruses (A and B not differentiated), RSV (A and B not differentiated), Cytomegalovirus (CMV), Adenovirus, Enterovirus and Parechovirus), 11 bacteria (*Streptococcus pneumoniae*, *Staphylococcus aureus*, *Haemophilus influenzae and Haemophilus* *influenzae* type B, *Moraxella cattarahalis*, *Legionella* spp., *Salmonella* spp., *Chlamydia pneumoniae*, *Mycoplasma pneumoniae*, *Klebsiella pneumoniae* and *Bordetella pertussis*) and the fungus *Pneumocystis jiroveci*. All samples were tested in the same country they were collected using standard operating procedures across all sites and standard curves were used to calculate pathogen load from PCR cycle threshold values [8].

Other tests included blood culture on cases, which were processed using the BacT/Alert microbial system (Organon Teknika, Durham, NC, USA) or the BD BACTEC FX200 blood culture system (BD Biosciences, Franklin Lakes, NJ, USA). Standard diagnostics were also conducted on all case blood samples to determine white blood cell counts (cases only) and C-reactive protein (CRP) levels (in cases and a subset of controls). Microbiologically confirmed pneumococcal pneumonia (MCPP) was defined as *Streptococcus pneumoniae* cultured from a normally sterile fluid. In addition, in the PERCH study a strong association was observed between high pneumococcal densities in the NP (>6.9 log_10_ copies/mL) [9] or whole blood (>2.2 log_10_ copies/mL) samples [10] and the presence of MCPP. Cases with high density pneumococcal (HDP) thresholds and/or MCPP were used to analyze the relationship between HCoVs and pneumococcal pneumonia.

### 2.3. Statistical Analysis

PCR pathogen loads were log_10_ transformed. Chi-squared and Wilcoxon tests were used to analyze the demographic characteristics of cases and controls. Binary, multinomial logistic regression and odds ratio analysis were used to model the prevalence of HCoV, *Betacoronavirus* and *Alphacoronavirus* within the study population. Binary and multinomial logistic regression analyses were used to model the prevalence of HCoV within the study population. Age categories and site of enrolment together with variables with an association at *p* < 0.2 in the univariate analysis were included in the multivariable models. All statistical analysis was performed using STATA Version 12.1 (College Station, TX, USA) and a two-sided *p*-value < 0.05 was considered statistically significant.

## 3. Results

Overall, community controls were more likely to have HCoV detected in their NP/OP samples compared to cases (10.5% vs. 7.2%; 95% CI: 1.17–1.96); however, this association was only evident in the asymptomatic controls (11% vs. 7.2%; 95% CI: 1.2–2.0) (Table 1); regardless of site of enrolment (Table S1 in Supplementary Material). Further, this association was seen in HIV-uninfected (HIV−) participants (10% vs. 7% for controls vs. cases) as well as in children living with HIV (HIV+; 10% vs. 7% for controls vs. cases). Interestingly, the prevalence of HCoV detection was similar in the cases and the RTI controls. However, the HIV+ and RTI analysis lacked statistical power due to the small number of HCoV positive participants.

The association with asymptomatic controls was driven by the *Betacoronavirus* endemic species (HKU1-HCoV and OC43-HCoV) and in particular HCoV-HKU1 (Table S2 in Supplementary Material), whereas the *Alphacoronavirus* endemic species (229E-HCoV and NL63-HCoV) were not associated with case or control status. Similar associations were only observed when the asymptomatic controls were compared to the cases (Table 1).

By age group, the association of HCoV detection being associated with asymptomatic controls was only evident in children > 6 months of age. There were no significant differences in the prevalence of HCoVs detected among HIV+ and HIV− cases and controls. Once again, the association of HCoV with controls > 6 months of age was driven by the *Betacoronavirus* endemic species (Table S3 in Supplementary Material).

HCoV was detected as the only respiratory virus present in the nasopharynx/oropharynx of significantly more of the asymptomatic controls than the cases. However, HCoV+ cases were more likely to have co-infections with RSV, HBoV and AdV compared to the HCoV+ asymptomatic controls. Additionally, compared to the HCoV+ asymptomatic controls the HCoV+ cases were more likely to have high density of pneumococcus (>6.9 log_10_ copies/mL) on NP/OP swabs. There were no differences in the prevalence of bacterial co-infections in the NP/OP of the HCoV+ cases and asymptomatic controls (Table 2). Similar trends were seen among the HIV− and HIV+ cases and controls, but the HIV+ analysis lacked statistical power to detect differences (Tables S4 and S5 in Supplementary Material).

**Figure 1:**
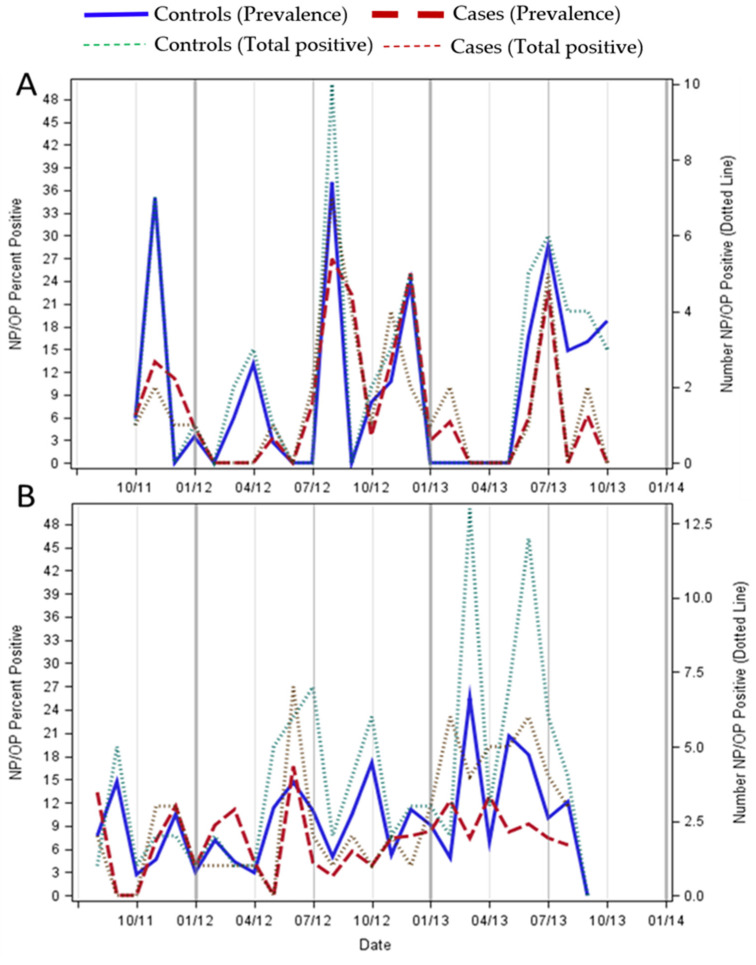
The seasonal distribution of HCoV over a period of two years in children hospitalized with pneumonia and asymptomatic controls in Zambia (Panel (**A**)) and South Africa (Panel (**B**)). Prevalence is the % of samples that tested positive for HCoV for each month for cases (dashed red line) and controls (solid blue line), with the actual number of HCoV positives for each month on the z axis for cases (dotted green line) and controls (dotted red line).

**Figure 2:**
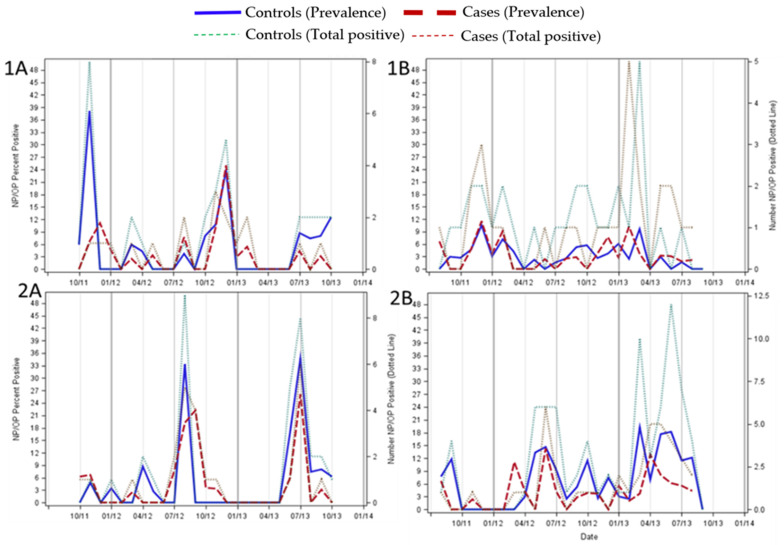
The seasonal distribution of *Alphacoronavirus* endemic species (Panel (**1A**) and (**1B**)) and *Betacoronavirus* endemic species (Panel (**2A**) and (**2B**)) over a period of two years in children hospitalized with pneumonia and asymptomatic controls in Zambia (Panel (**1A**) and (**2A**)) and South Africa (Panel (**1B**) and (**2B**)). Prevalence is the % of samples that tested positive for HCoV for each month for cases (dashed red line) and controls (solid blue line), with the actual number of HCoV positives for each month on the z axis for cases (dotted green line) and controls (dotted red line).

In Zambia, HCoV infections in both cases and asymptomatic controls occurred mainly during the winter months—July to September. However, in South Africa the seasonal distribution was less well defined with cases and controls having different peaks in prevalence’s (Figure 1). Among the *Alphacoronavirus* endemic species, HCoV-NL63 and -229E, the peaks tended to occur later in the year in the warmer months (November-January); whereas the *Betacoronavirus* endemic species, HCoV-OC43 and -HKU1, tended to peak in the colder months (May–August; Figure 2).

Among children with pneumonia, HCoV was not associated with very severe pneumonia; there was however a trend for the *Betacoronavirus* endemic species to be more associated with very severe pneumonia and longer hospital stays (>5 days) compared to HCoV- cases. Additionally, the *Betacoronavirus* endemic species were more likely to have medically significant CRP levels (>40 mg/mL), high density of pneumococcus (>6.9 log_10_ copies/mL) on NP/OP swabs and the HCoV+ cases were more likely to have blood cultures positive for medically significant bacteria compared to HCoV- cases (Table 3). This association with blood culture positivity was mainly driven by cases were HCoV was the only respiratory virus detected in the nasopharynx whereas the cases where HCoV was detected together with other common respiratory viruses were more likely to be hypoxic (Table S6 in Supplementary Material).

There was however no difference in the prevalence of common bacteria in the NP/OP of HCoV+ cases compared to HCoV- cases. Compared to HCoV- cases, the HCoV+ cases were more likely to be co-infected with HBoV but less likely to be co-infected with Parainfluenza viruses (Table 3).

**Figure 3:**
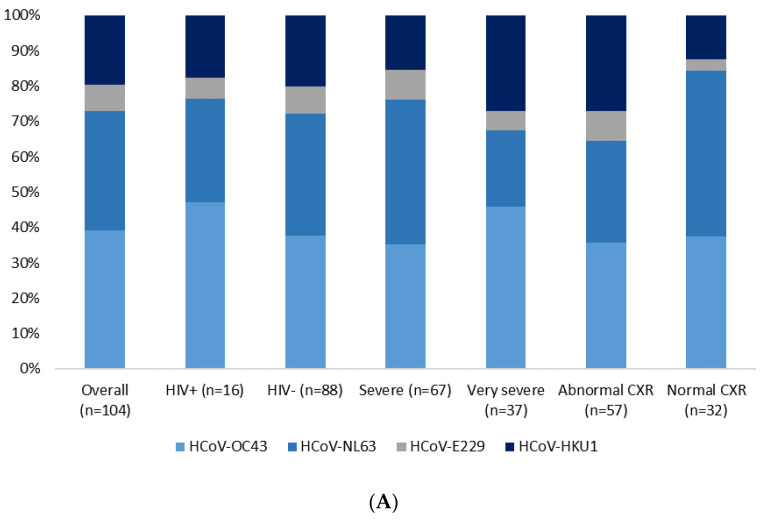

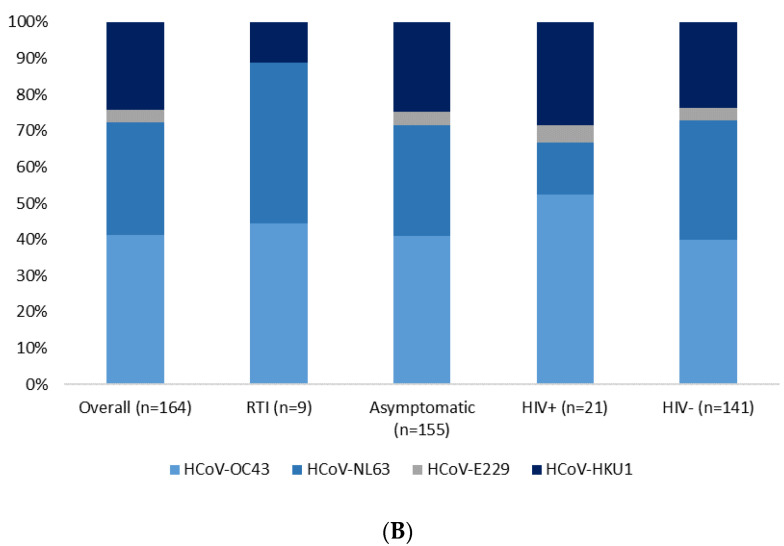
Distribution of HCoV species among HCoV positive cases (Panel (**A**)) and controls (Panel (**B**)).

There were no differences in the distribution of HCoV species by HIV status or clinical presentation of pneumonia. Similarly, among the controls, there were no differences in the distribution of HCoV by HIV status or presence of RTI symptoms (Figure 3). Further, HCoV detection was not associated with the presence of RTI symptoms or co-infections with other viral pathogens (Table S7 in Supplementary Material).

## 4. Discussion

In this multi-country, case–control pneumonia etiology study reporting on the epidemiology of the four endemic HCoV infections in children under the age of 5 years, we found that HCoV detection was more prevalent in children living in the community compared to children hospitalized with pneumonia. Interestingly, this association was only evident in the asymptomatic children compared to hospitalized children. Among the children with RTI, the prevalence of HCoV detection was similar to that of the hospitalized children; albeit the numbers in this group were small. This association with asymptomatic controls was, however, only observed among children > 6 months and was mainly driven by the *Betacoronavirus* endemic species (HCoV-OC43 and –HKU1). The prevalence of HCoV detection reported in this study among the cases (7%) was similar to those reported in previous studies including in Africa [2,3,4,11,12,13,14]. Further, the prevalence of *Alphacoronaviruses* reported in this study is similar to other studies conducted in South Africa reporting on CoV-NL63 detection in sick children [12,15,16]. The prevalence of HCoV detection reported in this study in asymptomatic children is substantially higher than reported in another South African study [16] (11% versus 0%); however, in the other South African study the sample size of healthy children was very limited (*n* = 46) compared to our study (*n* > 1500).

Among controls, the prevalence of HCoV detection was not associated with signs and symptoms of mild respiratory tract infections, nor was it associated with HIV status. Similarly, among cases, HCoV detection was not associated with very severe pneumonia, signs and symptoms of more severe disease, or HIV status. However, *Betacoronavirus* detection among cases showed a trend to be associated with more severe pneumonia as well as longer hospital stays. HCoV, and in particular *Betacoronavirus,* detection, was also associated with blood culture positivity, medically significant CRP levels and high pneumococcal densities in the NP/OP samples, all indicators of bacterial co-infection. This concurs with reports which have suggested that bacterial infections play an essential part in the pathogenesis of some viral infections progressing to severe respiratory disease [17,18]. Further, the HCoV positive cases, in particular *Betacoronaviruses*, were positively associated with Human bocavirus detection compared to the HCoV negative cases potentially resulting in a more severe infection [19].

Further, similar to previous studies [12,14,15,16,20], mixed infections with other respiratory viruses was common among the cases, whereas the asymptomatic controls were more likely to only have HCoV detected; thus, the mixed viral infections could have predisposed the children to more severe disease or the detection of HCoV in the cases was incidental to the severe disease outcome. Several studies have found that higher viral loads are associated with more severe disease [21,22,23,24] but in this study there were no differences in the viral loads between the HCoV positive cases and asymptomatic controls thus potentially further indicating that the HCoV detection in cases was incidental to the severe disease outcome. From SARS-CoV-2 studies we also know that HCoV can shed for up to 3 weeks [25] after infection took place thus detection might not be indicative of a current infection and our study used URT sampling as a proxy for sampling the site of infection. Direct sampling of the LRT, including lung aspirates and bronchoalveolar lavage, would provide more direct evidence on the causal pathogen of the pneumonia episode.

Several studies have looked at the seasonality of HCoV [11,15,16,26,27,28,29]. However, the majority of these are in high income countries in the Northern Hemisphere which have very defined seasons. To our knowledge, this is the first study reporting on the seasonality of HCoV in both hospitalized children and asymptomatic controls in the Southern Hemisphere where the seasons are more temperate. In both Zambia and South Africa, HCoV was detected throughout the year. However, in Zambia the peak of infections tended to occur during the colder months (June to September); which was mainly driven by the *Betacoronaviruses*. Whether such transmission peaks apply to the current circulation of SARS-CoV-2, part of the *Betacoronavirus* genera, in Southern Hemisphere countries has yet to be determined.

Even though Zambia and South Africa have high HIV rates in the general population (12.5% and 20%, respectively) [6]; mother to child transmission prevention strategies have likely reduced HIV rates in children [30] (<14% in our population). Thus, we lacked statistical power to study the clinical epidemiology of HCoV in HIV positive children living in South Africa and Zambia. However, it did appear that HIV was not a risk factor for more severe HCoV disease with HIV positive asymptomatic controls having a higher prevalence of HCoV detection than the HIV positive cases. Only one other case–control study looked at HCoV detection in a high HIV population [16]; however, due to the even more limited control enrolment they too failed to study the risk factor of HIV and HCoV. Other limitation included the cross-sectional design which limited our ability to study the temporal association of HCoV and timing of disease onset and controls were not followed up after enrolment into the study to determine if they went on to develop disease. Additionally, our study used upper respiratory tract sampling as a proxy for actual site of infection, namely, the lower respiratory tract. Direct sampling would provide better evidence for the actual causal agent of the pneumonia episode; these samples are however invasive and difficult to perform.

## 5. Conclusions

In conclusion, HCoV and in particular endemic *Betacoronaviruses* were more commonly detected in asymptomatic children, compared to children requiring hospitalization with severe pneumonia. Further, there was little evidence that HCoVs were directly contributing to the severe disease outcome observed in this study. However, co-infections with other common respiratory viruses, namely human bocavirus, and bacteria cannot be dismissed when it comes to childhood disease with more studies required to fully elucidate the etiologic contribution of HCoV.

## Figures and Tables

**Table 1:** Number of study subjects enrolled and tested for HCoV—percent positive by age and HIV-1 infection status.

Age Group			Total N (%)	HIV+ N (%)	HIV− N (%)	
Overall	Enrolled	Cases	1537	218	1316	
		Asymptomatic controls	1485	170	1306	
		RTI Controls	165	51	113	
	HCoV ^g^	Cases	104 (7)	16 (7)	88 (7)	
		Controls	164 (11)	21 (10)	141 (10)	*p* = 0.569 ^f^
			***p* = 0.002 ^c^**	*p* = 0.385 ^c^	***p* = 0.002 ^c^**	
		Asymptomatic control ^a^	155 (11)	18 (11)	135 (11)	*p* = 0.357 ^f^
			***p* = 0.001 ^d^**	*p =* 0.320 ^d^	***p* = 0.001 ^d^**	
		RTI control ^b^	9 (6)	3 (7)	6 (6)	*p =* 0.167 ^f^
			*p* = 0.698 ^e^	*p* = 0.817 ^e^	*p* = 0.738 ^e^	
	AlphaCoV ^h^	Cases	44 (3)	6 (3)	38 (3)	
		Controls	59 (4)	4 (2)	54 (4)	*p* = 0.336 ^f^
			*p* = 0.232 ^c^	*p* = 0.362 ^c^	*p* = 0.140 ^c^	
		Asymptomatic control ^a^	55 (4)	1 (2)	51 (4)	*p* = 0.258 ^f^
			*p* = 0.194 ^d^	*p* = 0.749 ^d^	*p* = 0.150 ^d^	
		RTI control ^b^	4 (2)	3 (2)	3 (3)	*p* = 0.538 ^f^
			*p* = 0.746 ^e^	*p* = 0.521 ^e^	*p* = 0.887 ^e^	
	BetaCov ^i^	Cases	63 (4)	11 (5)	52 (4)	
		Controls	111 (7)	17 (8)	92 (6)	*p* = 0.922 ^f^
			***p* = 0.002 ^c^**	*p* = 0.244 ^c^	***p* = 0.040 ^c^**	
		Asymptomatic control ^a^	106 (7)	2 (4)	89 (7)	*p* = 0.217 ^f^
			***p* = 0.002 ^d^**	*p* = 0.736 ^d^	***p* = 0.001 ^d^**	
		RTI control ^b^	5 (3)	15 (9)	3 (3)	*p* = 0.477 ^f^
			*p* = 0.505 ^e^	*p* = 0.140 ^e^	*p* = 0.492 ^e^	

Abbreviations: n: number; HCoVs: Human coronaviruses; HIV: Human immunodeficiency virus; RTI: Respiratory tract infections. a—Asymptomatic controls were controls presenting with no obvious signs or symptoms of respiratory tract infections at the time of sample collection. b—Controls were considered to have RTI if they had (1) cough or runny nose, or (2) one of the following signs, ear discharge, wheeze, or difficulty breathing, in the presence of sore throat or fever (temperature ≥ 38.0 °C or reported fever in the past 48 h). c—*p*-values adjusted for age in months and site of enrolment where applicable, comparing the HCoV prevalence among cases and controls. d—*p*-values adjusted for age in months and site of enrolment where applicable, comparing the HCoV prevalence among cases and asymptomatic controls. e—*p*-values adjusted for age in months and site of enrolment where applicable, comparing the HCoV prevalence among cases and RTI controls. f—*p*-value adjusting for age in months and site of enrolment where applicable, comparing the HCoV prevalence HIV+ and HIV− cases and controls. g—any HCoV species (OC43 and/or NL43 and/or E229 and/or HKU1). h—*Alphacoronavirus,* HKU1-HCoV and OC43-HCoV. i—*Betacoronavirus,* 229E-HCoV and NL63-HCoV.

**Table 2:** HCoV among cases (*n* = 104) and asymptomatic controls (*n* = 164) at South African and Zambian sites.

Characteristics	Cases N (%)	Asymptomatic Controls N (%)	aOR (95%CI)	*p*-Value ^a^
HCoV epidemiology				
HCoV detected as single viral infections ^b^	38 (36)	89 (57)	2.64 (1.55–4.53)	**<0.001**
HCoV-E229	8 (8)	6 (4)	0.49 (0.16–1.49)	0.206
HCoV-NL63	36 (35)	49 (32)	0.91 (0.53–1.56)	0.730
HCoV-OC43	42 (40)	66 (43)	1.07 (0.63–1.81)	0.802
HCoV-HKU1	21 (20)	40 (26)	1.32 (0.72–2.44)	0.367
HCoV load, mean (SD) ^d^	5.29 (0.77)	5.29 (0.68)	-	0.973
Load in HCoV single infections	5.43 (0.27)	5.33 (0.17)	-	0.77
Load in HCoV mixed infections	5.18 (0.22)	5.23 (0.20)	-	0.87
Mixed viral infections in the NP/OP				
Two HCoV species ^c^	3 (3)	2 (1)	0.59 (0.22–2.45)	0.39
RSV	24 (23)	2 (1)	0.05 (0.01–0.21)	**<0.001**
InFV (A, B and C)	4 (4)	2 (1)	0.22 (0.03–1.40)	0.108
AdV	14 (13)	10 (6)	0.34 (0.14–0.82)	**0.017**
HBoV	19 (18)	17 (11)	0.47 (0.22–0.98)	**0.045**
RV	20 (19)	28 (18)	0.92 (0.48–1.76)	0.806
PIV (1–4)	2 (2)	4 (3)	1.26 (0.21–7.40)	0.797
Bacterial co-infections in the NP/OP				
*H. influenzae* type b	5 (5)	2 (1)	0.24 (0.05–1.33)	0.104
*B. pertussis*	2 (2)	1 (1)	0.41 (0.04–4.72)	0.473
*S. aureus*	21 (20)	23 (15)	0.86 (0.43–1.71)	0.658
*H. influenzae*	52 (50)	73 (47)	0.79 (0.47–1.33)	0.377
*S. pneumoniae*	74 (71)	114 (74)	1.09 (0.61–1.95)	0.773
*M. pneumoniae*	1 (1)	3 (2)	1.27 (0.12–13.5)	0.840
*M. catarrhalis*	71 (68)	116 (75)	1.39 (0.79–2.47)	0.257
*C. pneumoniae*	0	5 (3)	-	0.082
HCoV and *S. pneumoniae* co-infections				
*S. pneu* load, mean (SD) ^e^	5.42 (0.16)	5.77 (1.37)	-	0.067
HDP in nasopharynx ^f^	15 (14)	11 (7)	2.46 (1.06–5.67)	**0.035**
*S. pneu* detected in WB	5 (5)	11 (7)	1.38 (0.45–4.25)	0.570

Abbreviations: HCoV: human coronavirus; aOR: adjusted odds ratio; CI: confidence interval; WB: whole blood; RV: rhinovirus; RSV: respiratory syncytial virus, HMPV: human metapneumovirus; PIV: parainfluenza types 1–4; HBoV: human bocavirus; AdV: adenovirus; InFV: influenza virus (A, B and C); *S. aureus: Staphylococcus aureus; S. pneu: Streptococcus pneumoniae*; *H. influenzae: Haemophilus influenzae, H. influenzae* type, *M. catarrhalis: Moraxella catarrahalis. B. pertussis: Bordetella pertussis; M. pneumoniae: Mycoplasma pneumoniae; C. pneumoniae: Chlamydia pneumoniae*; HDP: High density pneumococcus. a—*p*-values and aOR from regression models adjusted for age in months, site of enrolment, and confounding covariates where applicable. b—Human Coronavirus including either OC43, NL63, 229E or HKU1. c—Human Coronavirus including OC43 and/or NL63 and/or 229E and/or HKU1. d—HCoV viral load in the nasopharynx expressed as log_10_ copies/mL. e—*S. pneumoniae* bacterial load in the nasopharynx expressed as log_10_ copies/mL. f—HDP defined as *S. pneumoniae* density in nasopharynx > 6.9 log_10_ copies/mL.

**Table 3:** Demographic, clinical characteristics and markers of bacterial and respiratory viral co-infections among severe and very-severe pneumonia cases identified with Human coronavirus infection.

Characteristics	HCoV ^a^ + (*n* = 104) N(%)	AlphaCoV ^b^ (*n* = 44) N(%)	BetaCoV ^c^ (*n* = 63) N(%)	HCoV- (*n* = 1338) N(%)	*p*-Value ^d^	*p*-Value ^e^	*p*-Value ^f^
Demographics and health							
Age (months), mean (SD)	7.9 (8.52)	9.07 (9.55)	7.51 (8.05)	8.9 (10.01)	0.337	0.917	0.276
Male	64 (62)	27 (61)	39 (62)	703 (52)	0.07	0.241	0.136
HIV	16 (15)	6 (14)	11 (17)	188 (14)	0.654	0.921	0.409
Clinical features							
Very severe pneumonia	37 (35)	10 (23)	27 (43)	428 (32)	0.463	0.204	0.08
Chest X-ray abnormal ^g^	57 (58)	22 (53)	37 (63)	693 (54)	0.583	0.712	0.26
Hypoxic ^h^	69 (67)	27 (63)	44 (70)	829 (62)	0.274	0.807	0.175
Tachycardia ^i^	59 (57)	29 (67)	33 (53)	737 (55)	0.696	0.111	0.606
Tachypnea ^j^	83 (82)	38 (86)	48 (79)	1081 (82)	0.999	0.439	0.607
Wheezing	24 (23)	11 (26)	14 (22)	321 (24)	0.948	0.785	0.855
Convulsions	2 (2)	1 (2)	1 (2)	38 (3)	0.613	0.793	0.582
Diarrhea	16 (15)	6 (14)	10 (16)	238 (18)	0.485	0.473	0.636
Hospital stay > 5 days	59 (57)	20 (45)	39 (62)	665 (50)	0.174	0.619	0.06
Died in Hospital	9 (9)	4 (9)	5 (8)	127 (9)	0.755	0.912	0.643
Bacterial co-infection markers							
Fever ^k^	74 (71)	30 (68)	46 (73)	963 (72)	0.947	0.573	0.782
Leukocytosis ^l^	593 (45)	51 (50)	19 (44)	34 (54)	0.302	0.925	0.122
CRP > 40 mg/L ^m^	35 (34)	12 (27)	25 (40)	381 (28)	0.186	0.848	0.034
Blood culture positive ^n^	8 (8)	3 (7)	5 (8)	47 (4)	**0.039**	0.266	0.084
MCPP ^o^	1 (1)	1 (2)	0	11 (1)	0.829	0.339	0.47
Bacterial co-infections							
*S. aureus*	21 (20)	11 (25)	10 (16)	333 (25)	0.291	0.911	0.111
*S. pneumoniae*	74 (71)	29 (66)	48 (76)	919 (68)	0.571	0.662	0.202
*M. catarrhalis*	71 (68)	26 (59)	47 (75)	850 (63)	0.314	0.500	0.071
*B. pertussis*	2 (2)	0	2 (3)	19 (1)	0.678	0.417	0.233
*H. influenzae*	52 (50)	18 (41)	37 (59)	669 (50)	0.977	0.226	0.152
*H. influenzae* type b	5 (5)	1 (2)	4 (6)	38 (3)	0.254	0.757	0.122
*M. pneumoniae*	1 (1)	0	1 (2)	7 (1)	0.561	0.615	0.258
*C. pneumoniae*	0	0	0	16 (1)	0.263	0.480	0.395
HDP in nasopharynx ^p^	15 (14)	3 (7)	13 (21)	144 (11)	0.245	0.436	**0.006**
HDP in blood ^q^	4 (4)	3 (7)	1 (2)	67 (5)	0.603	0.529	0.222
Respiratory viral co-infections in the Nasopharynx							
RSV	24 (23)	7 (16)	18 (29)	320 (24)	0.76	0.234	0.478
AdV	14 (13)	6 (14)	9 (14)	130 (10)	0.165	0.375	0.172
HMPV	3 (3)	2 (5)	2 (3)	94 (7)	0.117	0.529	0.249
HBoV	19 (18)	8 (18)	12 (19)	152 (11)	**0.024**	0.166	**0.045**
InFV A-C	4 (4)	2 (5)	2 (3)	59 (5)	0.618	0.861	0.546
PIV	2 (2)	1 (2)	1 (2)	138 (10)	**0.014**	0.117	**0.05**
RV	20 (19)	6 (14)	15 (24)	303 (23)	0.460	0.167	0.770

Abbreviations: SD: standard deviation; CRP: C-reactive protein; MCPP: microbiologically confirmed pneumococcal pneumonia; *S. aureus: Staphylococcus aureus; S. pneu: Streptococcus pneumoniae*; *H. influenzae: Haemophilus influenzae, H. influenzae* type, *M. catarrhalis: Moraxella catarrahalis. B. pertussis: Bordetella pertussis; M. pneumoniae: Mycoplasma pneumoniae; C. pneumoniae: Chlamydia pneumonia;* RSV: respiratory syncytial virus, HMPV: human metapneumovirus; AdV: adenovirus; PIV: parainfluenza type 1–4; HBoV: human bocavirus; HCoV: human coronavirus (OC43, NL63, 229E and HKU1); RV: rhinovirus; and InFV: influenza virus (A, B and C). a—HCoV includes cases positive for any of the four endemic species (NL63, OC43, HKU1 and/or 229E). b—AlphaCoV includes cases positive for NL63 and/or 229E. c—BetaCoV includes cases positive for OC43 and/or HKU1. d—*p*-values from regression models comparing HCoV positive compared to HCoV negative cases adjusted for age in months, site of enrollment and for confounding covariates where applicable where applicable. e—*p*-values from regression models comparing AlphaHCoV species (NL63 and 229E) positive compared to HCoV negative cases adjusted for age in months, site of enrolment and for confounding covariates where applicable. f—*p*-values from regression models comparing BetaHCoV species (OC43 and HKU1) positive compared to HCoV negative cases adjusted for age in months, site of enrolment and for confounding covariates where applicable. g—Abnormal Chest X-ray defined as radiographically confirmed end point pneumonia consolidation or any infiltrates. h—A child was considered to be hypoxic if (1) a room air pulse-oximetry reading indicated oxygen saturation < 90%, or (2) a room air oxygen saturation was not available and child was placed on supplemental oxygen. i—Tachycardia defined as heart rate > 160 beats per minute (bpm) if aged < 11 months, heart rate > 150 bpm if aged 12–35 months, heart rate > 140 bpm if aged 36–59 months. j—Tachypnea defined as respiratory rate ≥ 60 breaths/minute if aged < 2 months, respiratory rate ≥ 50 breaths/minute if aged 2–12 months, respiration rate ≥ 40 breaths/minute if aged > 12 month. k—Fever defined as temperature ≥ 38 °C. l—Leukocytosis defined as white blood cell count > 15,000 cells/uL if age < 12 months or >13,000 cells/uL if age > 12 months. m—CRP defined as levels ≥ 40 mg/L were considered to potentially indicate bacterial infection. n—Blood culture positive for any non-contaminating bacteria. o—MCPP defined as *S. pneumoniae* cultured from a normally sterile body fluid, including blood, pleural fluid or lung aspirate, or as pleural fluid or lung aspirate positive on PCR *LytA* testing. p—HDP defined as *S. pneumoniae* density in nasopharynx > 6.9 log_10_ copies/mL. q—HDP defined as *S.pneumoniae* density in whole blood > 2.2 log_10_ copies/mL.

## Data Availability

All data is available at https://clinepidb.org/ce/app (accessed on 12 May 2021).

## Supplementary Materials

The following are available online at https://www.mdpi.com/article/10.3390/v13081513/s1, Table S1: Number of study subjects enrolled and tested for HCoV—percent positive by site of enrolment, Table S2: Number of study subjects enrolled and tested for the four endemic HCoV species, Table S3: Number of study subjects enrolled and tested for HCoV—percent positive by age and HIV-1 infection status, Table S4: Characteristics of HCoV+ in HIV+ cases (N = 16) and controls (N = 21), Table S5: Characteristics of HCoV+ in HIV− cases (N = 88) and controls (N = 141), Table S6: Demographic, clinical characteristics and markers of bacterial and respiratory viral co-infections among severe and very-severe pneumonia cases identified with Human coronavirus infection, Table S7: Demographic, clinical characteristics and markers of bacterial and respiratory viral co-infections among community controls identified with Human coronavirus infection.
