# Supplementary material for: Epidemiology and Seasonality of Endemic Human Coronaviruses in South African and Zambian Children: A Case-Control Pneumonia Study
Source: Viruses. 2021 Jul 31;13(8):1513. doi: 10.3390/v13081513 (PMC8402793; doi:10.3390/v13081513)
Supplement: Supplementary file 1 [file viruses-13-01513-s001.zip › viruses-1284699-supplementary.pdf]

Table S1: Number of study subjects enrolled and tested for HCoV - percent positive by site of enrolment.

Site	N (%)		Total	HIV+	HIV-
Soweto, South Africa	Enrolled	Cases	920	115	805
		Controls	964	136	822
	HCoV[f]	Cases	65 (7)	5 (4)	60 (8)
		Controls	104 (11)	15 (11)	88 (11)
			***p*=0.005[c]**	*p*=0.051[c]	***p*=0.026[c]**
		Asymptomatic control[a]	101 (11)	15 (11)	85 (11)
			***p*=0.001[d]**	***p*=0.037[d]**	***p*=0.018[d]**
		RTI control[b]	3 (6)	0	3 (7)
			p=0.698[e]	*p*=0.547[e]	*p*=0.826[e]
Lusaka, Zambia	Enrolled	Cases	617	103	511
		Controls	686	85	597
	HCoV[f]	Cases	39 (7)	11 (12)	28 (6)
		Controls	60 (11)	6 (8)	53 (10)
			p=0.113[c]	*p*=0.377[c]	***p*=0.033[c]**
		Asymptomatic control[a]	54 (10)	3 (8)	50 (10)
			p=0.069[d]	*p*=0.462[d]	***p*=0.021[d]**
		RTI control[b]	6 (7)	3 (8)	3 (6)
			p=0.864[e]	*p*=0.518[e]	*p*=0.892[e]

Abbreviations: n: number; HCoVs: Human coronaviruses; HIV: Human immunodeficiency virus; RTI: Respiratory tract infections

a – Asymptomatic controls were controls presenting with no obvious signs or symptoms of respiratory tract infections at the time of sample collection

b – Controls were considered to have RTI if they had 1) cough or runny nose, or 2) one of the following signs, ear discharge, wheeze, or difficulty breathing, in the presence of sore throat or fever (temperature ≥38.0°C or reported fever in the past 48 hours).

c - *p*-values adjusted for age in months comparing the HCoV prevalence among cases and controls.

d - *p*-values adjusted for age in months comparing the HCoV prevalence among cases and asymptomatic controls.

e - *p*-values adjusted for age in months comparing the HCoV prevalence among cases and RTI controls.

f - any HCoV species (OC43 and/or NL63 and/or E229 and/or HKU1).

Table S2: Number of study subjects enrolled and tested for the four endemic HCoV species.

N (%)	Cases (n=1447)	RTI controls[a] (n=142)	Asymptomatic controls[b] (n=1424)	P-value[c]	P-value[d]
NL63	42 (3)	4 (3)	66 (5)	0.954	0.110
E229	8 (1)	0	6 (0.5)	0.374	0.613
HKU1	21 (1)	1 (1)	40 (3)	0.467	**0.017**
OC43	36 (2)	4 (3)	49 (3)	0.811	0.132

Abbreviations: n: number; HCoVs: Human coronaviruses; HIV: Human immunodeficiency virus; RTI: Respiratory tract infections

a – Asymptomatic controls were controls presenting with no obvious signs or symptoms of respiratory tract infections at the time of sample collection

b – Controls were considered to have RTI if they had 1) cough or runny nose, or 2) one of the following signs, ear discharge, wheeze, or difficulty breathing, in the presence of sore throat or fever (temperature ≥38.0°C or reported fever in the past 48 hours).

c - *p*-values adjusted for age in months and site on enrolment comparing the HCoV prevalence among cases and RTI controls.

d - *p*-values adjusted for age in months and site of enrolment comparing the HCoV prevalence among cases and asymptomatic controls.

Table S3: Number of study subjects enrolled and tested for HCoV - percent positive by age and HIV-1 infection status.

Age group			Total N (%)	HIV+ N (%)	HIV- N (%)	
1-5 months	Enrolled	Cases	741	94	647	
		Controls	636	65	566	
	HCoV[c]	Cases	59 (8)	8 (9)	51 (8)	
		Controls	66 (10)	6 (9)	59 (10)	*p*=0.41[b]
			p=0.123[a]	*p*=0.773[a]	*p*=0.127[a]	
	AlphaCoV[d]	Cases	23 (3)	1 (1)	22 (3)	
		Controls	29 (5)	1 (2)	28 (5)	p=0.639[b]
			p=0.174[a]	*p*=0.560[a]	*p*=0.199[a]	
	BetaCov[e]	Cases	37 (5)	7 (7)	30 (5)	
		Controls	38 (6)	5 (8)	32 (6)	*p*=0.717[b]
			p=0.410[a]	*p*=0.945[a]	*p*=0.398[a]	
6-11 month	Enrolled	Cases	357	57	300	
		Controls	420	58	360	
	HCoV[c]	Cases	23 (6)	3(5)	20 (7)	
		Controls	45 (11)	4 (7)	41 (11)	*p*=0.703[b]
			***p*=0.038[a]**	*p*=0.625[a]	***p*=0.038[a]**	
	AlphaCoV[d]	Cases	9 (3)	1 (2)	8 (3)	
		Controls	17 (4)	1 (2)	16 (4)	*p*=0.501[b]
			p=0.242[a]	*p*=0.859[a]	*p*=0.228[a]	
	BetaCov[e]	Cases	14 (4)	2 (4)	12 (4)	
		Controls	31 (7)	3 (5)	28 (8)	*p*=0.735[b]
			p=0.043[a]	*p*=0.648[a]	*p*=0.045[a]	
12-59 months	Enrolled	Cases	349	53	295	
		Controls	510	87	420	
	HCoV[c]	Cases	22 (6)	5 (10)	17 (6)	
		Controls	53 (10)	11 (13)	41 (10)	*p*=0.917[b]
			***p*=0.038[a]**	*p*=0.561[a]	*p*=0.054[a]	
	AlphaCoV[d]	Cases	12 (3)	4 (8)	8 (3)	
		Controls	13 (2)	2 (2)	10 (2)	*p*=0.539[b]
			p=0.429[a]	*p*=0.157[a]	*p*=0.768[a]	
	BetaCov[e]	Cases	12 (3)	2 (4)	10 (3)	
		Controls	42 (8)	9 (10)	32 (8)	*p*=0.504[b]
			***p*=0.005[a]**	*p*=0.177[a]	***p*=0.020[a]**	

Abbreviations: n: number; HCoVs: Human coronaviruses; HIV: Human immunodeficiency virus; RTI: Respiratory tract infections.

a - *p*-values adjusted for age in months and site of enrolment where applicable, comparing the HCoV prevalence among cases and controls.

b – p-value adjusting for age in months and site of enrolment where applicable, comparing the HCoV prevalence HIV+ and HIV- cases and controls.

c - any HCoV species (OC43 and/or NL43 and/or E229 and/or HKU1).

d – *Alphacoronavirus*, HKU1-HCoV and OC43-HCoV.

e – *Betacoronavirus*, 229E-HCoV and NL63-HCoV.

Table S4: Characteristics of HCoV+ in HIV+ cases (N=16) and controls (N=21)

Characteristics	Cases N(%)	Controls N(%)	aOR (95%CI)	*p*-value[a]
HCoV detected as single viral infections[b]	8 (50)	14 (67)	0.27 (0.05-1.47)	0.13
HCoV-OC43	8 (50)	11 (52)	1.05 (0.22-5.03)	
HCoV-NL63	5 (31)	3 (14)	4.15 (0.53-32.2)	
HCoV-E229	1 (5)	1 (6)	0.33 (0.12-8.96)	0.513
HCoV-HKU1	3 (19)	6 (29)	0.52 (0.08-3.25)	0.484
Mixed infections with:				
Two HCoV species[c]	1 (6)	0		0.245
RSV	0	1 (%)	-	0.376
RV	3 (19)	3 (14)	1.21 (0.15-10.1)	0.855
PIV (1-4)	0	0	-	-
HBoV	5 (31)	4 (19)	7.81 (0.80-76.1)	0.077
AdV	3 (19)	0		**0.049**
InFV (A,B and C)	0	0	-	-
HCoV load, mean (SD)[d]	5.09 (1.43)	5.58 (1.60)	-	0.35
S. pneu co-detected in NP/OP	15 (94)	114 (67)	13.90 (1.51-168)	**0.038**
S. pneu load, mean (SD)[e]	5.79 (1.51)	5.98 (1.03)	-	0.692
HDP in nasopharynx[f]	6 (38)	2 (10)	17.24 (1.31-227)	**0.03**
S. pneu detected in WB	1 (7)	1 (5)	1.42 (0.08-24.8)	0.806
HDP in blood[g]	0	0	-	-

Abbreviations: HCoV: Human coronavirus; aOR: adjusted odds ratio; CI: Confidence interval; WB: whole blood; RV: Rhinovirus; RSV: Respiratory Syncytial Virus, HMPV: Human Metapneumovirus; PIV: Parainfluenza type 1-4; HBoV: Human Bocavirus; AdV: Adenovirus; InFV: Influenza Virus (A, B and C); *S. pneu*: *Streptococcus pneumoniae*; HDP: High density pneumococcus.

a - *P*-values and aOR from regression models adjusted for age in months, site of enrolment, and confounding covariates where applicable.

b – Human Coronavirus including either OC43, NL63, 229E or HKU1.

c - Human Coronavirus including OC43 and/or NL63 and/or 229E and/or HKU1.

d - HCoV viral load in the nasopharynx expressed as log_{10} copies/mL.

e – *S. pneumoniae* bacterial load in the nasopharynx expressed as log_{10} copies/mL.

f - HDP defined as *S. pneumoniae* density in nasopharynx >6.9 log_{10} copies/mL.

g – HDP defined as *S.pneumoniae* density in whole blood >2.2 log_{10} copies/mL.

Table S5: Characteristics of HCoV+ in HIV- cases (N=88) and controls (N=141)

Characteristics	Cases N(%)	Controls N(%)	aOR (95%CI)	*p*-value[a]
HCoV detected as single viral infections[b]	30 (34)	80 (57)	0.35 (0.20-0.64)	**<0.001**
HCoV-OC43	34 (39)	57 (40)	0.91 (0.51-1.61)	0.735
HCoV-NL63	31 (35)	49 (35)	0.94 (0.53-1.68)	0.844
HCoV-E229	7 (8)	5 (4)	2.59 (0.77-8.63)	0.122
HCoV-HKU1	18 (20)	35 (25)	0.86 (0.44-1.66)	0.653
Mixed infections with:				
Other HCoV type[c]	2 (2)	5 (4)	0.68 (0.12-3.66)	0.649
RSV	24 (27)	1 (1)	49.58 (6.49-379)	**<0.001**
RV	17 (19)	27 (19)	0.97 (0.48-1.94)	0.928
PIV (1-4)	2 (2)	5 (4)	0.65 (0.12-3.64)	0.623
HBoV	14 (16)	14 (10)	2.16 (0.94-4.97)	0.071
AdV	11 (13)	11 (8)	2.10 (0.84-5.32)	0.116
InFV (A,B and C)	4 (5)	2 (1)	5.14 (0.77-34.5)	0.092
HCoV load, mean (SD)[d]	5.32 (1.83)	5.25 (.71)	-	0.773
S. pneu co-detected in NP/OP	59 (67)	108 (77)	0.68 (0.36-1.27)	0.225
S. pneu load, mean (SD)[e]	5.33 (1.32)	5.73 (1.18)	-	**0.034**
HDP in nasopharynx[f]	9 (10)	9 (6)	1.67 (0.624.49)	0.308
S. pneu detected in WB	4 (5)	10 (7)	0.63 (0.18-2.15)	0.455
HDP in blood[g]	0	0	-	-

Abbreviations: HCoV: Human coronavirus; aOR: adjusted odds ratio; CI: Confidence interval; WB: whole blood; RV: Rhinovirus; RSV: Respiratory Syncytial Virus, HMPV: Human Metapneumovirus; AdV: adenovirus; PIV: Parainfluenza type 1-4; HBoV: Human Bocavirus; AdV: Adenovirus; InFV: Influenza Virus (A, B and C); *S. pneu*: *Streptococcus pneumoniae*; HDP: High density pneumococcus.

a - *P*-values and aOR from regression models adjusted for age in months, site of enrolment, and confounding covariates where applicable.

b – Human Coronavirus including either OC43, NL63, 229E or HKU1.

c - Human Coronavirus including OC43 and/or NL63 and/or 229E and/or HKU1.

d - HCoV viral load in the nasopharynx expressed as $\log_{10}$ copies/mL.

e – *S. pneumoniae* bacterial load in the nasopharynx expressed as $\log_{10}$ copies/mL.

f - HDP defined as *S. pneumoniae* density in nasopharynx >6.9 $\log_{10}$ copies/mL.

g – HDP defined as *S.pneumoniae* density in whole blood >2.2 $\log_{10}$ copies/mL.

Table S6. Demographic, clinical characteristics and markers of bacterial and respiratory viral co-infections among severe and very-severe pneumonia cases identified with Human coronavirus infection.

Characteristics	Mono HCoV[a]+ (n=38) N(%)	Mixed HCoV[b] (n=66) N(%)	HCoV- (n=1338) N(%)	*p*-value[c]	*p*-value[d]
Demographics and health:					
Age (months), mean (SD)	6.5 (1.36)	8.8 (1.05)	8.9 (10.01)	0.148	0.898
Male	24 (63)	40 (61)	703 (52)	0.176	0.194
HIV	8 (21)	8 (12)	188 (14)	0.292	0.643
Clinical features:					
Very severe pneumonia	11 (29)	26 (39)	428 (32)	0.682	0.206
Chest X-ray abnormal[e]	19 (51)	38 (62)	693 (54)	0.314	0.248
Hypoxic[f]	19 (51)	50 (76)	829 (62)	0.253	**0.033**
Tachycardia[g]	23 (61)	36 (55)	737 (55)	0.688	0.847
Tachypnea[h]	31 (84)	52 (80)	1081 (82)	0.703	0.793
Wheezing	6 (16)	18 (27)	321 (24)	0.630	0.780
Convulsions	1 (3)	1 (2)	38 (3)	0.983	0.577
Diarrhea	7 (18)	9 (14)	238 (18)	0.906	0.356
Died in Hospital	7 (23)	2 (4)	127 (9)	0.551	0.070
Fever[i]	32 (84)	42 (63)	963 (72)	0.072	0.171
Leukocytosis[j]	52 (55)	30 (46)	34 (54)	0.130	0.866
Blood culture positive[k]	5 (13)	3 (5)	47 (4)	**0.004**	0.639
MCPP[l]	1 (3)	0	11 (1)	0.140	0.459
Bacterial co-infections:					
S. aureus	8 (21)	13 (20)	333 (25)	0.351	0.353
S. pneumoniae	28 (74)	46 (70)	919 (68)	0.443	0.785
M. catarrhalis	26 (68)	45 (68)	850 (63)	0.370	0.417
B. pertussis	2 (5)	0	19 (1)	0.143	0.330
H. influenzae	17 (45)	35 (53)	669 (50)	0.936	0.721
H. influenzae type b	3 (8)	2 (3)	38 (3)	0.081	0.869
M. pneumoniae	0	1 (2)	7 (1)	0.655	0.317
C. pneumoniae	0	0	16 (1)	0.498	0.372
HDP in nasopharynx[m]	5 (13)	10 (15)	144 (11)	0.631	0.274
HDP in blood[n]	0	0	67 (5)	-	-

Abbreviations: SD: standard deviation; CRP: C-reactive protein; MCPP: microbiologically confirmed pneumococcal pneumonia; *S. aureus: Staphylococcus aureus; S. pneu: Streptococcus pneumoniae; H. influenzae: Haemophilus influenzae, H. influenzae* type, *M. catarrhalis: Moraxella catarrahalis. B. pertussis: Bordetella pertussis; M. pneumoniae: Mycoplasma pneumoniae; C. pneumoniae: Chlamydia pneumonia;*

a – HCoV (NL63, OC43, HKU1 and/or 229E) was the only respiratory virus detected in the nasopharynx.

b – HCoV (NL63, OC43, HKU1 and/or 229E) was detected as a mixed infection in the nasopharynx with other common respiratory viruses.

c - *P*-values from regression models comparing HCoV+ mono-infections compared to HCoV negative cases adjusted for age in months, site of enrollment and for confounding covariates where applicable where applicable.

d - *P*-values from regression models comparing HCoV+ mixed infections compared to HCoV negative cases adjusted for age in months, site of enrolment and for confounding covariates where applicable.

e – Abnormal Chest X-ray defined as radiographically confirmed end point pneumonia consolidation or any infiltrates.

f - A child was considered to be hypoxic if 1) a room air pulse-oximetry reading indicated oxygen saturation <90%, or 2) a room air oxygen saturation was not available and child was placed on supplemental oxygen.
g - Tachycardia defined as heart rate >160 beats per minute (bpm) if aged <11 months, heart rate >150 bpm if aged 12-35 months, heart rate >140 bpm if aged 36-59 months.
h - Tachypnea defined as respiratory rate ≥60 breaths/minute if aged <2 months, respiratory rate ≥50 breaths/minute if aged 2-12 months, respiration rate ≥40 breaths/minute if aged >12 month.
i - Fever defined as temperature ≥38°C.
j - Leukocytosis defined as white blood cell count >15 000 cells/uL if age <12 months or >13 000 cells/uL if age >12 months.
k - Blood culture positive for any non-contaminating bacteria.
l - MCPP defined as *S. pneumoniae* cultured from a normally sterile body fluid, including blood, pleural fluid or lung aspirate, or as pleural fluid or lung aspirate positive on PCR *LytA* testing.
m - HDP defined as *S. pneumoniae* density in nasopharynx >6.9 $\log_{10}$ copies/mL.
n – HDP defined as *S.pneumoniae* density in whole blood >2.2 $\log_{10}$ copies/mL.

Table S7: Demographic, clinical characteristics and markers of bacterial and respiratory viral co-infections among community controls identified with Human coronavirus infection

Characteristics	HCoV[a]+ (n=164) N(%)	AlphaCoV[b] (n=59) N(%)	BetaCoV[c] (n=111) N(%)	HCoV- (n=1338) N(%)	*p*-value[d]	*p*-value[e]	*p*-value[f]
Demographics and health							
Age (months), mean (SD)	11.84(12.3)	9.52(10.1)	13.1 (12.9)	11.96	0.904	0.127	0.317
Male	73 (45)	30 (51)	46 (41)	697 (50)	0.207	0.776	0.098
HIV	21 (13)	4 (7)	17 (15)	189 (13)	0.601	0.182	0.195
Clinical features:							
RTI control	9 (5)	4 (7)	5 (5)	133 (9)	0.118	0.401	0.095
Fever	2 (1)	0	2 (2)	16 (1)	0.930	0.386	0.557
Cough	5 (3)	2 (3)	3 (3)	64 (5)	0.534	0.808	0.461
Rhinitis	2 (1)	2 (3)	0	42 (3)	0.365	0.942	0.125
Ear discharge	0	0	0	7 (1)	0.558	0.832	0.697
Vomiting	0	0	0	3 (0)	0.706	0.896	0.806
Diarrhoea	2 (1)	1 (2)	1 (1)	12 (1)	0.749	0.794	0.896
Respiratory viral co-infections in the NP/OP:							
RSV	2 (1)	1 (2)	1 (1)	49 (4)	0.492	0.491	0.147
AdV	11 (7)	3 (5)	8 (7)	119 (8)	0.434	0.361	0.665
HMPV	4 (2)	1 (2)	3 (3)	43 (3)	0.757	0.549	0.848
HBoV	18 (11)	1 (2)	17 (15)	143 (10)	0.757	0.065	0.099
InFV A-C	2 (1)	0	2 (2)	34 (2)	0.330	0.243	0.777
PIV	5 (3)	3 (5)	2 (2)	69 (5)	0.285	0.233	0.051
RV	30 (18)	11 (19)	21 (19)	289 (21)	0.485	0.737	0.694
Bacterial co-infections:							
S. aureus	23 (14)	8 (14)	16 (15)	225 (16)	0.514	0.624	0.698
S. pneumoniae	122 (75)	44 (75)	83 (76)	1005(72)	0.417	0.671	0.421
M. catarrhalis	125 (77)	45 (76)	86 (78)	953 (68)	**0.025**	0.219	0.031
B. pertussis	1 (1)	1 (2)	0	3 (0)	0.340	**0.026**	0.582
H. influenzae	78 (48)	23 (39)	58 (53)	615 (44)	0.344	0.398	0.066
H. influenzae type b	3 (2)	0	3 (3)	22 (2)	0.784	0.322	0.322
M. pneumoniae	3 (2)	0	3 (3)	3 (0)	**0.002**	0.627	**0.001**
C. pneumoniae	5 (3)	0	5 (5)	18 (1)	0.074	0.339	**0.005**
HDP in nasopharynx[g]	11 (7)	3 (5)	1 (2)	121 (9)	0.402	0.393	0.396
HDP in blood[h]	6 (4)	1 (2)	5 (5)	54 (4)	0.903	0.396	0.668

Abbreviations: SD: standard deviation; RSV: respiratory syncytial virus, HMPV: human metapneumovirus; AdV: adenovirus; PIV: parainfluenza type 1-4; HBoV: human bocavirus; HCoV: human coronavirus (OC43, NL63, 229E and HKU1); RV: rhinovirus; and InFV: influenza virus (A, B and C). *S. aureus: Staphylococcus aureus; S. pneu: Streptococcus pneumoniae; H. influenzae: Haemophilus influenzae, H. influenzae* type, *M. catarrhalis: Moraxella catarrahalis. B. pertussis: Bordetella pertussis; M. pneumoniae: Mycoplasma pneumoniae; C. pneumoniae: Chlamydia pneumoniae.*

a – HCoV includes cases positive for any of the four endemic species (NL63, OC43, HKU1, 229E).

b – AlphaCoV includes cases positive for NL63 and/or 229E.

c – BetaCoV includes cases positive for OC43 and/or HKU1.

d - *P*-values from regression models comparing HCoV positive compared to HCoV negative cases adjusted for age in months, site of enrollment and for confounding covariates where applicable.
e - *p*-values from regression models comparing AlphaHCoV species (NL63 and 229E) positive compared to HCoV negative cases adjusted for age in months, site of enrolment and for confounding covariates where applicable.
f - *p*-values from regression models comparing BetaHCoV species (OC43 and HKU1) positive compared to HCoV negative cases adjusted for age in months, site of enrolment and for confounding covariates where applicable.
g - HDP defined as *S. pneumoniae* density in nasopharynx >6.9 log_{10} copies/mL.
h – HDP defined as *S.pneumoniae* density in whole blood >2.2 log_{10} copies/mL.
